# Prevalence and Evolution of Noroviruses between 1966 and 2019, Implications for Vaccine Design

**DOI:** 10.3390/pathogens10081012

**Published:** 2021-08-11

**Authors:** Hong-Lu Zhou, Li-Na Chen, Song-Mei Wang, Ming Tan, Chao Qiu, Tian-Yi Qiu, Xuan-Yi Wang

**Affiliations:** 1 Key Laboratory of Medical Molecular Virology of MoE & MoH and Institutes of Biomedical Sciences, Fudan University, Shanghai 200032, China; 16111510020@fudan.edu.cn (H.-L.Z.); 20111510055@fudan.edu.cn (L.-N.C.); qiuchao@fudan.edu.cn (C.Q.); 2 Laboratory of Molecular Biology, Training Center of Medical Experiments, School of Basic Medical Sciences, Fudan University, Shanghai 200032, China; smwang2@fudan.edu.cn; 3 Division of Infectious Diseases, Cincinnati Children’s Hospital Medical Center, Cincinnati, OH 45229, USA; ming.tan@cchmc.org; 4 College of Medicine, University of Cincinnati, Cincinnati, OH 45229, USA; 5 Zhong Shan Hospital, Shanghai Public Health Clinical Center, Shanghai Medical College, Fudan University, Shanghai 201508, China; 6 Children’s Hospital, Fudan University, Shanghai 200062, China; 7 Shanghai Institute of Infectious Disease and Biosecurity, Shanghai 200032, China

**Keywords:** norovirus, prevalence, evolution, homology modeling, immunogen

## Abstract

Noroviruses (NoVs), a group of single-stranded RNA viruses causing epidemic acute gastroenteritis in humans, are highly diverse, consisting of multiple genogroups with >30 genotypes. Their continual evolutions make NoV vaccine design and development difficult. Here, we report a study of NoV sequences obtained from a population-based diarrhea surveillance in Zhengding County of Hebei Province spanning from 2001 to 2019 and those available in the GenBank database from 1966 to 2019. NoV genotypes and/or variants that may evade immunity were screened and identified based on primary and conformational structures for vaccine design. We selected 366, 301, 139, 74 and 495 complete VP1-coding nucleotide sequences representing the predominant genotypes of GII.4, GII.2, GII.3, GII.6 and GII.17, respectively. A total of 16 distinct GII.4 variants were identified, showing a typical linear evolutionary pattern of variant replacement, while only 1–4 variants of the other genotypes were found to co-circulate over the 40–50-year period without typical variant replacement. The vaccine strain GII.4c is close to variant Sydney_2012 (0.053) in their primary structure, but they are distinct at epitopes A and E in conformations. Our data suggested GII.4 variant Sydney_2012, GII.2 variant A, a GII.3 strain, GII.6 variants B and C and GII.17 variant D are primary candidate strains for NoV vaccine development.

## 1. Introduction

Noroviruses (NoVs) are a common cause of acute gastroenteritis among young children worldwide [1]. They are RNA viruses constituting the *Norovirus* genus in the family Caliciviridae. Each NoV virion is encapsulated by an icosahedral protein capsid containing a positive-sense, single-stranded RNA genome in ~7.7 kb with three open reading frames (ORFs) [2]. ORF1 encodes six non-structural proteins, while ORF2 and ORF3 encode the major (VP1) and the minor (VP2) structural proteins, respectively. Structure studies revealed that VP1 has two major regions, a N-terminal shell (S) and a C-terminal protruding (P) domain. The latter is further divided into the moderately conservative P1 and the highly variable P2 subdomains [3,4]. In vitro expression of VP1 results in self-assembled virus-like particles (VLPs), being composed of 90 VP1 dimers.

A standardized nomenclature has recently been proposed to classify NoVs into seven genogroups (GI–GVII) with at least 30 genotypes, based on the complete VP1 amino acid sequence [4]. Of these, genogroups GI and GII with 9 and 22 genotypes, respectively, primarily infect humans. Despite this extensive genetic diversity, genotype GII.4 is responsible for the majority of NoV outbreaks and epidemics worldwide [5]. However, two exceptions were noted recently, in which a GII.17 stain became predominant in East Asia during 2014–2015, and a GII.2 stain was predominant in Europe and Asia during 2016–2017 [6,7,8,9,10].

There is no commercial NoV vaccine so far. The fact that NoVs are diverse with multiple genogroups/genotypes circulating in human populations simultaneously and their continuous evolution make immunogen selection of a NoV vaccine difficult [2]. Although four vaccine candidates have entered clinical trials [11,12,13], different methods to tentatively select immunogens are still being debated. Thus, further molecular analyses of NoV evolution will shed light onto the design and development of NoV vaccines. Most of such previous studies focused on GenBank database or hospital-based surveillance [14,15,16], which may not reflect real evolutionary trends in nature. Therefore, a long time span, population-based surveillance in a particular locality are essential to examine the evolutionary dynamics of NoVs.

In this study, NoV genomic information was not only collected from GenBank database, but also from a population-based diarrhea surveillance. Through the orderly applications of epidemiology, bioinformatics and structural biology, a comprehensive understanding of the epidemiological characteristics, evolutionary patterns and structural changes of NoVs was achieved. Our findings suggested that non-GII.4 intra-genotypic variants displayed a striking genetic stability over long periods of time, with GII.4 as the notable exception, providing new insights into preliminary screening of candidate vaccine strains.

## 2. Results

### 2.1. Global Temporal Dynamics of NoVs

**Figure 1:**
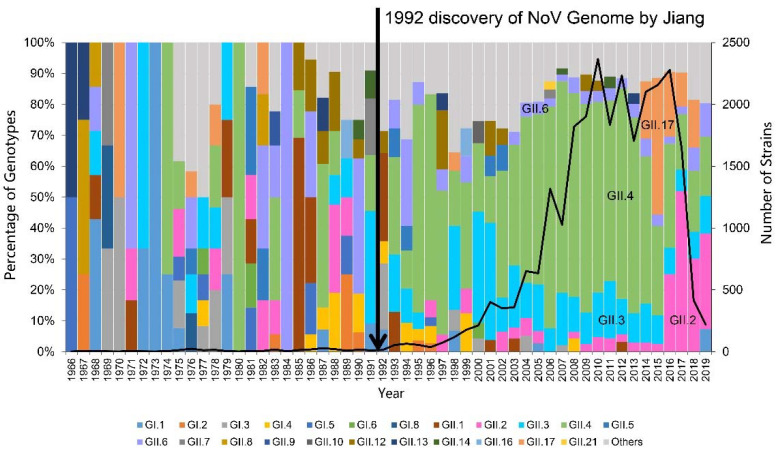
Global temporal dynamics of the prevalence of various norovirus genotypes over a period between 1966 and 2019 (*n* = 26,469). The percentage of the top five genotypes each year was presented, and the remaining genotypes are calculated as others.

To reveal the global temporal dynamics of NoVs, 26,469 NoV sequences with confirmed capsid types, including 362 from our population-based surveillance in Zhengding Country, and 26,107 available in the GenBank database were collected to analyze the prevalence of NoV genotypes during the past 50 years (Figure 1). In the early years (1966 to 1991), an epidemic trend was difficult to observe, due to the fewer number of available sequences. The accessible sequences in the GenBank started to increase rapidly since 1992, when the first PCR method was established to detect NoVs. Particularly, more than 1000 NoV sequences per year were deposited in the GenBank over the period from 2006 to 2017. From 2000 to 2013, the most prevalent genotype was GII.4, followed by GII.3. A sudden prevalence of GII.17 occurred in 2014, even surpassing GII.4 as the predominant genotype in 2015. An unexpected prevalence increase of GII.2 was also noted starting in 2016, and it has become the predominant genotype in recent years (2016–2019). In addition, although the prevalence of GII.6 was not as remarkable as the abovementioned ones, it has been steadily circulating as one of the top five epidemic genotypes since 2003. This global temporal dynamics indicates that the top five predominantly circulating genotypes are GII.4, GII.2, GII.3, GII.6 and GII.17. As a result, we began to further analyze their complete VP1 genes.

### 2.2. Time-Scale Evolution of the Globally Collected NoV Strains

A total of 2009 complete VP1-encoding nucleotide sequences of GII.4 were obtained from 1974 to 2019. To reduce the number of sequences while maintaining enough phylogenetic information, 366 of these sequences were selected based on criteria that at least one of each identified variant per country per year was included (Supplementary Table S1). Considering that fewer sequences (<500 sequences per genotype) were available for non-GII.4 genotypes, all available sequences were used in the analysis. As results, 301, 139, 74 and 495 sequences were derived for genotypes GII.2, GII.3, GII.6 and GII.17, respectively (Table 1).

**Figure 2:**
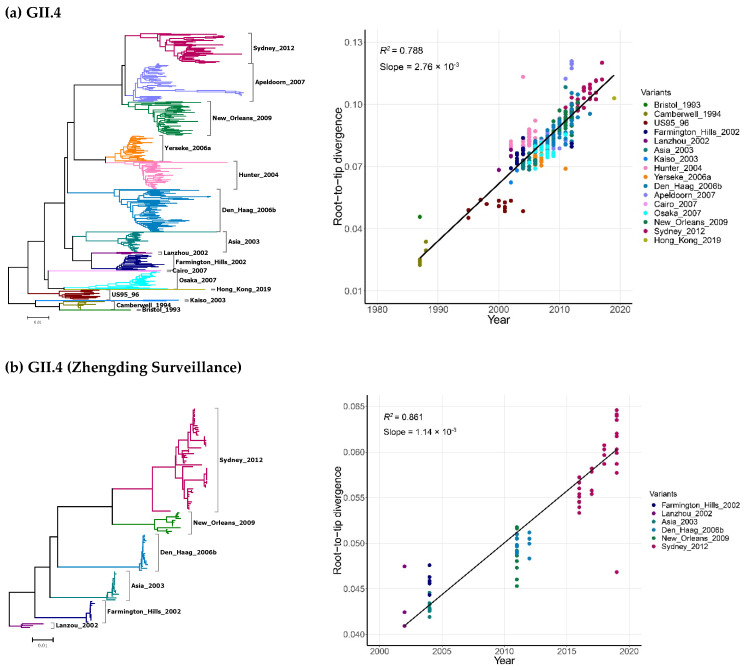

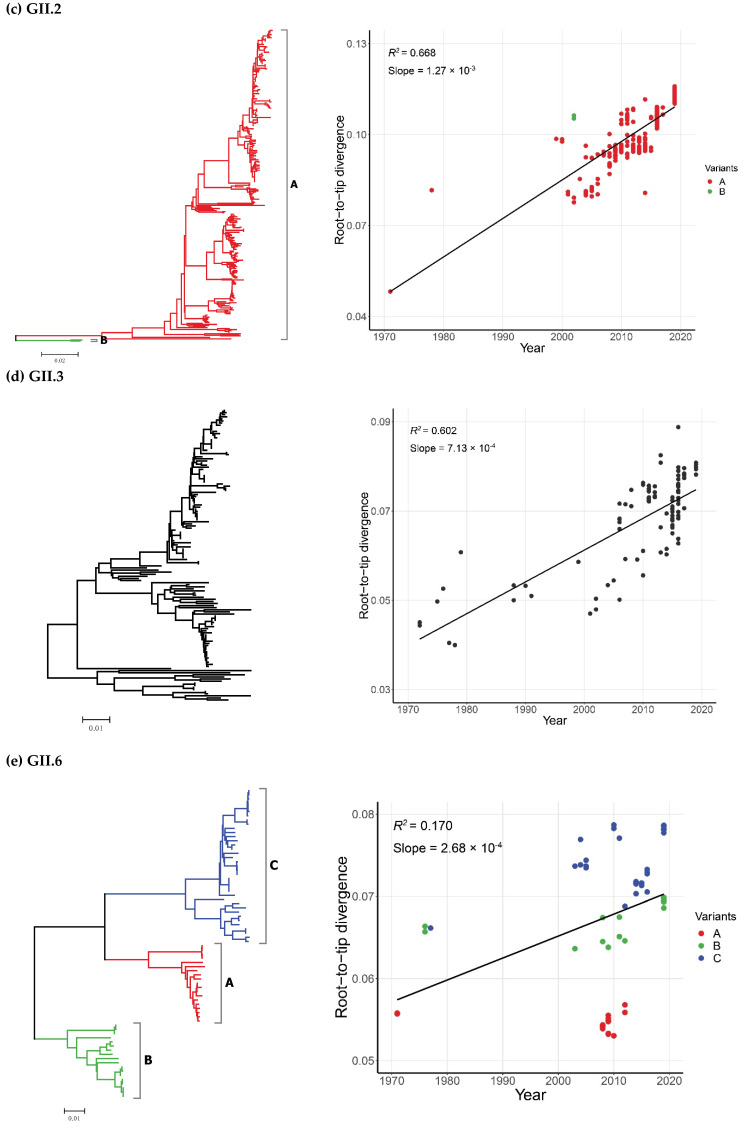

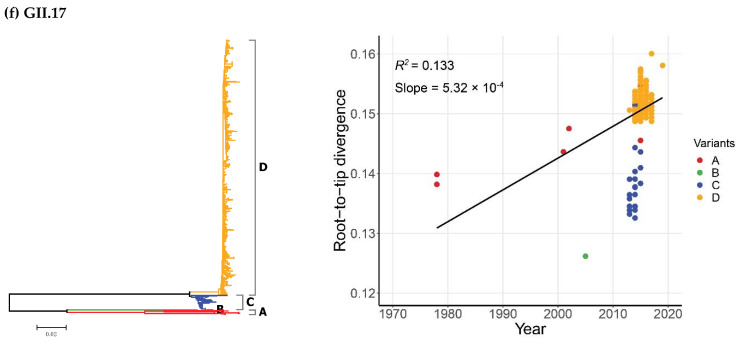
Phylogenetic trees and the corresponding root-to-tip divergence plots of the complete capsid genes (ORF2) of noroviruses. VP1-encoding nucleotide sequences of GII.4 (**a**), GII.4 from Zhengding surveillance (**b**), GII.2 (**c**), GII.3 (**d**), GII.6 (**e**) and GII.17 (**f**) genotypes are shown. The x-axis indicates the collection years, and the y-axis shows the root-to-tip divergence on the neighbor joining phylogenetic tree. The black line indicates a linear regression line of the root-to-tip divergence and collection years. Each circle presents a strain, and each color indicates a specific variant.

The GII.4 NoVs have been known to accumulate nucleotide substitutions in a clock-like manner for more than 30 years and diverged into 16 variants, showing periodic substitution of dominant variants. Presently, the main epidemic GII.4 strain is variant Sydney_2012 (Figure 2a). Considering the source of sequences from the GenBank was mixed and confounding, the variation rules of GII.4 were verified by the sequences collected from our long-term longitudinal and multi-sectional diarrhea surveillance in Zhengding County, Hebei Province (Figure 2b). Strikingly, it showed a similar evolutionary trend, confirming the consistency and reliability of the outcomes of the above analyses.

Unlike the GII.4 NoVs, the dominant variants in other genotypes appeared persist and co-circulate for 40–50 years without replacement or turnover of variants (Figure 2c–f). For example, GII.2 contained two variants, A and B. While 299 out of 301 sequences were from variant A that circulated from 1971 to 2019, only two sequences were variant B that emerged in 2002. Notably, only a single variant existed in GII.3 that circulated from 1972 to 2019. Three GII.6 variants (A, B, and C) were found to co-circulate since the 1970s, but only B and C existed after 2012. Finally, four GII.17 variants (A to D) were identified, among which variant A circulated over the longest period ranging from 1978 to 2016, but variant D has been the most prevalent one in recent years. The phylogenetic root-to-tip divergence plot showed a strong clock-like signal with coefficient of determination (R^2^) between 0.602 and 0.788 for GII.4, GII.2 and GII.3 genotypes, while GII.6 and GII.17 appeared to lack such clock-like evolution with R^2^ between 0.133 and 0.170.

To reconstruct the time-scale of each NoV genotype, the complete VP1 gene sequences were used for Bayesian coalescent analysis (Table 2). The results showed that the nucleotide substitution rates were estimated highest using UCED, followed by UCLN and strict clock, regardless of genotypes. When compared in the same molecular model, the highest nucleotide substitution rate was estimated in genotype GII.4 (4.95–5.91 × 10^−3^ nucleotide substitutions/site/year), followed by GII.3 (3.40–3.59 × 10^−3^ nucleotide substitutions/site/year), GII.2 (2.30–3.16 × 10^-3^ nucleotide substitutions/site/year), GII.6 (2.39–3.07 × 10^−3^ nucleotide substitutions/site/year), and GII.17 (1.54–1.98 × 10^−3^ nucleotide substitutions/site/year). The dates of the most recent ancestor could be traced back to 1791.9–1868.6 for GII.17, then 1855.6–1897.0 for GII.6, 1930.7–1945.5 for GII.2, 1967.1–1968.0 for GII.3 and 1980.5–1981.4 for GII.4. Notably, when GII.4 was confined to the same area (Zhengding County of our surveillance), the nucleotide substitution rate appeared to be lower, with 1.55–2.97 × 10^−3^ nucleotide substitutions/site/year.

**Figure 3:**
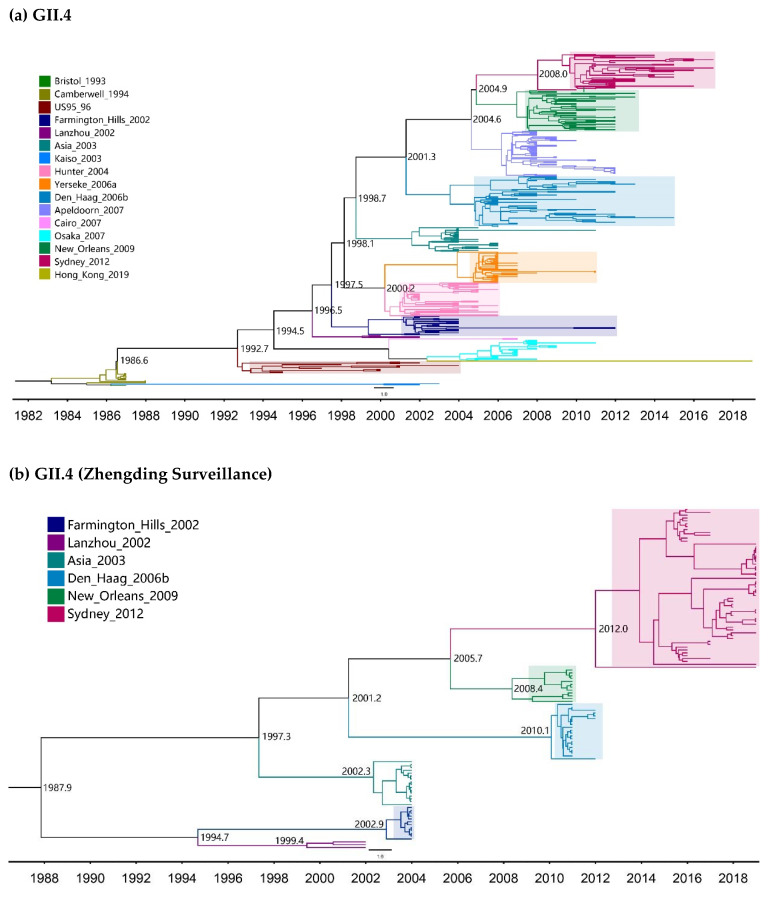

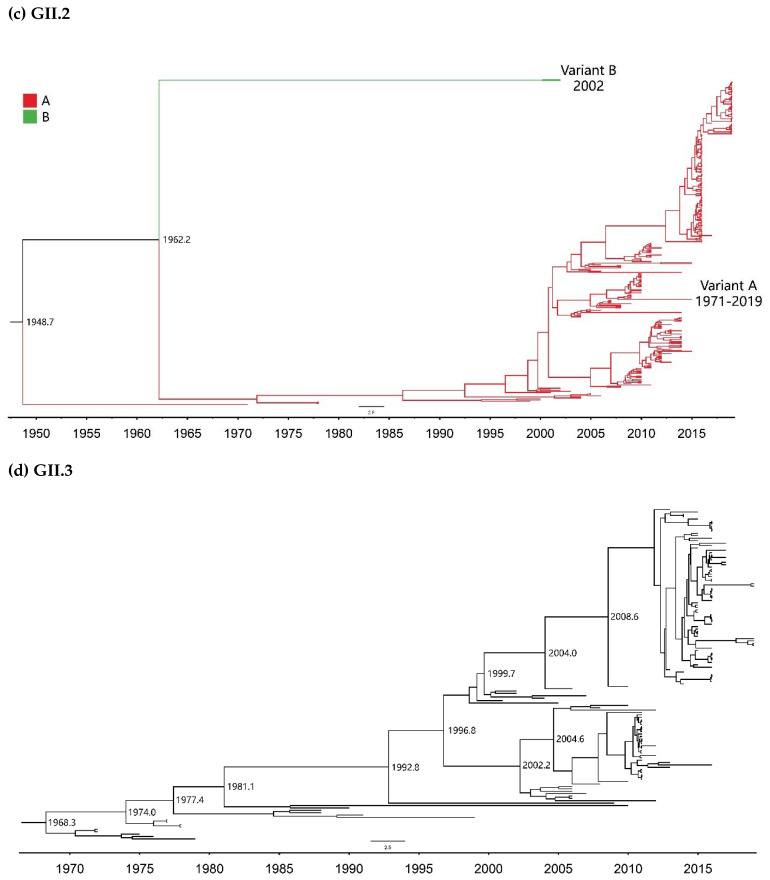

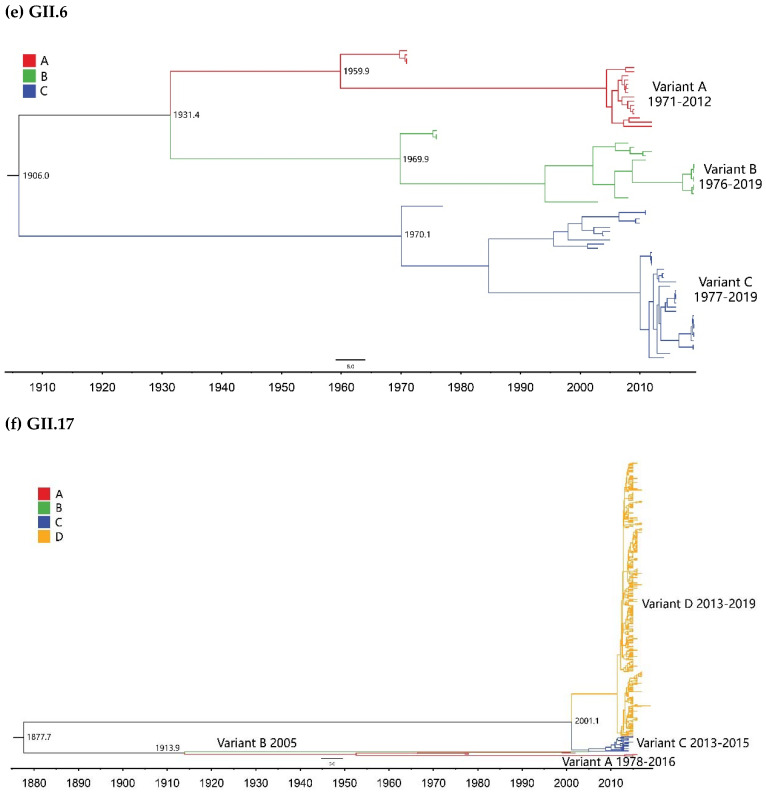
MCC tree based on complete capsid-encoding genes (ORF2s) of noroviruses. VP1-encoding nucleotide sequences of GII.4 (**a**), GII.4 from Zhengding surveillance (**b**), GII.2 (**c**), GII.3 (**d**), GII.6 (**e**) and GII.17 (**f**) genotypes. The trees were made using GTR + G + I nucleotide substitution model, an uncorrelated exponential clock (UCED) model and the Bayesian skyline as a tree prior. Each variant is shown by a specific color. A pandemic emergence of GII.4 is represented by the shaded area.

In addition, these Bayes factor values served as evidence that UCED was the best fit model for assessing the evolutionary history of the VP1 genes. Therefore, the MCC tree of complete VP1 genes was estimated by UCED using a Bayesian skyline demographic model (Figure 3). The results showed that GII.4 variants diverge from each other long before emerging to spread pandemically, indicating ongoing undetected circulation of pre-pandemic variants at a low level (Figure 3a,b). For the non-GII.4 genotypes, variant B of GII.2 that transiently emerged in 2002 may have started to diverge from variant A in 1962.2 (Figure 3c). Interestingly, GII.3 NoVs have been diverging among themselves with slight cumulative changes over time, although a new variant has not yet emerged (Figure 3d). Moreover, one branch of GII.6 began to diverge into variant C in 1906.0, and the other branch diverged into variants A and B in 1931.4 (Figure 3e). The divergent time of GII.17 variant C and D were estimated to be 2001.1 (Figure 3f).

### 2.3. Genetic Distances Based on Amino Acid of Complete VP1s

At present, the main epidemic strains of NoVs are Sydney_2012 of GII.4, variant A of GII.2, genotype GII.3 and variant B and C of GII.6, as well as variant D of GII.17. The mean amino acid distances among these main epidemic strains and the vaccine strain GII.4c of a vaccine candidate that is currently under a phase IIb clinical trial [17] were calculated (Table 3). The vaccine strain GII.4c is closest to Sydney_2012 of GII.4 (0.053) but further away from the other epidemic strains of heterologous genotypes (0.319–0.354). This raises concerns about the protective efficacy of the vaccine candidate against NoVs of heterologous genotypes.

### 2.4. Evolution of the GII.4 Blockade Epitopes

**Figure 4:**
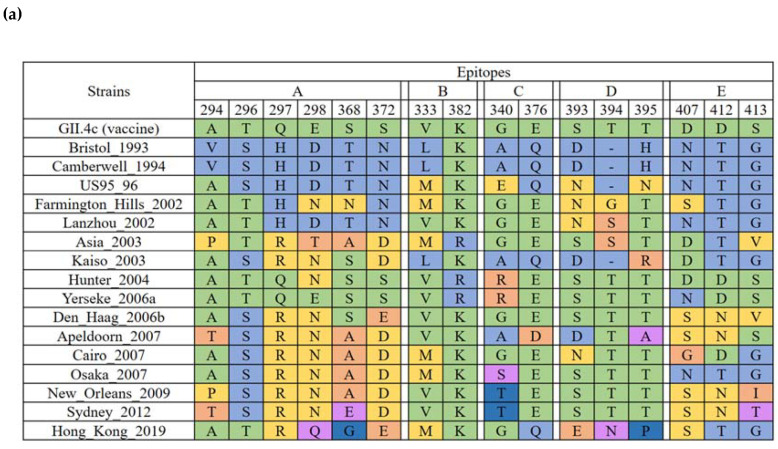

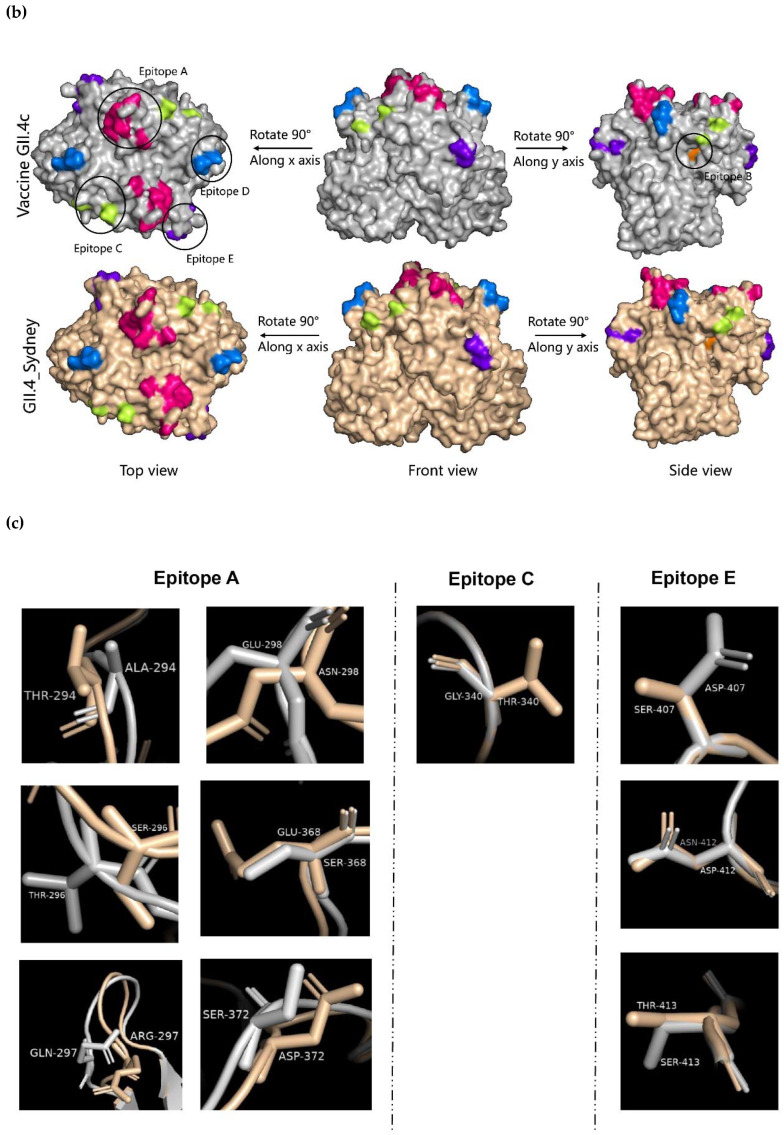
Predicted GII.4 norovirus evolving blockade epitopes. Bioinformatic approaches predicted five antibody epitopes on the surface of GII.4 noroviruses that appeared to be evolving over time and to correlate with the emergence of new GII.4 outbreak strains. (**a**) Amino acid variation of Epitopes A–E by GII.4 variant. (**b**) Surface representation of Sydney_2012 (brown) and GII.4c (grey). Epitope A (plum red), Epitope B (orange), Epitope C (lemon yellow), Epitope D (blue) and Epitope E (purple) are mapped onto P-dimer. (**c**) Cartoon representation of the subtle changes of blockade epitopes.

Previous studies have identified five blockade epitopes in VP1 of GII.4, referred as epitope A (294, 296–298, 368, 372), B (333, 382), C (340, 376), D (393–395) and E (407, 412–413), respectively [18]. By mapping the amino acid compositions of each variant over the past three decades (Figure 4a), we found that the five epitopes changed dynamically over time. For example, the amino acid at position 298 of epitope A was an aspartic acid (ASP, D) in variants Bristol_1993, Camberwell_1994 and US95_96. This residue mutated into an asparagine (Asn, N) in variant Farmington Hill_2002 that emerged in 2002 and remained the same until it was replaced by a glutamine (Gln, Q) in new variant, HongKong_2019, which emerged in 2019. It should be noted that aspartic acid is a negatively changed acidic amino acid, while asparagine is neutral, indicating a substantial property change. Other examples, including mutations Q376E (epitope C), D393N (epitope D) and N407D (epitope E), may also represent significant physic–chemical changes that could lead to critical alteration of the corresponding epitopes (Figure 4a).

Another potential substantial change of the epitopes could be caused by the deletion at site 394 in the four early variants of Bristol_1993, Camberwell_1994, US95_96 and Kaiso_2003. Finally, the epitopes A and E of the currently predominant variant Sydney_2012 differed completely from those of the vaccine strain GII.4c, while one amino acid mutation was also found in epitope C (Figure 4b,c). These substantial differences could potentially result in antigenicity drifting events between the two GII.4 variants, raising concerns on the protective efficacy of the vaccine candidate against the currently predominant GII.4 variant Sydney_2012.

## 3. Discussion

In this study, we analyzed and demonstrated the molecular evolution patterns of the predominant NoV genotypes, including GII.4, GII.2, GII.3, GII.6 and GII.17 based on their complete sequences of capsid protein VP1-encoding genes collected from NoVs that circulated over the past 40–50 years. Due to the known epochal evolution [19], novel GII.4 variants that cause epidemics or pandemics have emerged every 2 to 3 years, replacing the previous variants, and spread rapidly in the human population. By contrast, the genotypes GII.2, GII.3 and GII.6 presented a relatively lower evolutionary rate with fewer variants that could persist and co-circulate over the past 40–50 years without frequent substitutions or turnovers of variants. These findings explain the epidemiological characteristics of various NoVs in human population, that is, GII.4 is the main epidemic genotype in adults, while GII.2, GII.3 and GII.6 NoVs circulate with GII.4 among children [20,21,22]. Owing to the long-term circulation of the same GII.2, GII.3 and GII.6 variants, the child would establish the effective immune barrier upon infection after birth; as a result, the GII.2, GII.3 and GII.6 genotypes are often seen among adults. In contrast, people are always susceptible to GII.4 NoVs due to frequent emergences of new variants [14]. Based on current understanding, the GII.4 persistence in human populations might be promoted by the antigenic drift, which is suggested by the variation in surface-exposed residues and in residues around the fucose ligand interaction domain [23]. Nevertheless, in the preliminary data from an ongoing birth cohort study conducted in infants, we did detect a few infants with longer asymptomatic shedding (>1 month) after clinical recovery. Thus, we might speculate boldly that these excessively long shedding events serve as possible reservoirs for the high recombination rate in the population. Certainly, considering the widespread existence of norovirus in humans and animals, it might be another source that is responsible for the recombination. However, recent studies [24,25] indicate that evidence for transmission of animal norovirus to humans is sparse; therefore, based on the current body of evidence, an association between animal norovirus and reservoir for humans could not be established at this moment.

Of note, the emergence of a novel GII.17 variant after long-term silence resulted in the short-term outbreaks in winter of 2014–2015. It was found that this new variant was more common in older children and adults. For instance, as described in a hospital-based diarrhea surveillance in Shanghai, GII.17 (51.2%, 22/43) was more detected in adults (>15 years old), while no GII.17 (0%, 0/35) was detected in children (<15 years old) [26]. In another study among the hospitalized cases of acute gastroenteritis in Hong Kong, the proportions of GII.17 infected individuals were 15.6%, 47.7% and 36.7% in the <5 years, 5–65 years and >65 years age groups, respectively [27]. A possible explanation for these observations was that there was greater competitive pressure on the new variant of GII.17 for young children due to the co-circulation of the GII.4, GII.2, GII.3 and GII.6 genotypes. However, the immune barrier had been established in older children and adults attributed to their childhood infection. Only the alternative GII.4 variant had a competitive relationship with new emerging GII.17 variant, which led to more chance of infection for GII.17.

At present, the cross-protection between genotypes/variants and whether a candidate vaccine will induce a broad spectrum of immunity remain unknown. The notion that epitopes A (294, 296–298, 368, 372) and E (407, 412–413) of the currently prevalent GII.4 variant Sydney_2012 are distinct from those of the vaccine strain GII.4c raises concerns about the potential lacking of cross-protection the candidate vaccine that is currently under phase IIb clinical trial [17]. Previous studies showed that epitope A likely formed a non-contiguous, conformational epitope that changed over time, suggesting its important role associated with immune escape [28]. On the other hand, epitope E is found in surface-exposed regions lateral to the histo-blood group antigens (HBGAs) binding pockets. The compositions of epitope E varied with every major epidemic variant after 2002, indicating that it was a hot spot for the emergence of immunologically novel GII.4 variants [18]. In the phase I clinical trial, cross-reactive IgG to GII.2, GII.3 and GII.14 VLPs were observed after vaccination of bivalent GII.1/GII.4c VLPs vaccine [29]. Moreover, a recent study has demonstrated that the levels of HBGA-blocking antibodies and Pan-Ig induced by the GII.4c VLPs were increased in some placebo acute gastroenteritis (AGE) cases infected with GII.2. These findings suggested the vaccine strain GII.4c may possess a certain degree of cross-protection to other genotypes within the same genogroup [17].

According to the estimations by molecular clock model, the mean evolutionary rate of GII.4 was 4.95 × 10^−3^–5.91 × 10^−3^ nucleotide substitutions/site/year, with emergences of 16 variants over the past several decades, which were consistent with those reported earlier studies (4.30 × 10^−3^–7.68 × 10^−3^ nucleotide substitutions/site/year) [30,31,32,33]. However, a lower rate (1.55 × 10^−3^–2.97 × 10^−3^ nucleotide substitutions/site/year) was observed in our NoV surveillance in Zhengding County. This finding suggested that evolution patterns may be conserved, overall, but they could also be different in a specific population in a specific area. It was noted that the new variant Hong_Kong_2019 that was first isolated from a female patient with AGEs in August 2019 shared 92.6% amino acid sequence homology with capsid of the variant Osaka_2007 (GenBank: GQ845369) [34]. Considering that variant Sydney_2012 has been predominant since 2012, the emergence of variant Hong_Kong_2019 may indicate a new round of variant replacement.

GII.2 NoVs were rarely reported worldwide before 2016, with a few exception cases in Japan [35,36]. This study showed a mean evolutionary rate of 2.30 × 10^−3^–3.16 × 10^−3^ nucleotide substitutions/site/year of GII.6 that was similar to that reported in previous studies (2.99 × 10^−3^–3.24 × 10^−3^ nucleotide substitutions/site/year) [14,37]. We noted that VP1 sequences of the currently prevalent GII.2 strain differed only slightly (~2.3%) compared with one circulated in the 1970s. Interestingly, the capsid sequence of the strain GII.P2/GII.2 that emerged in 2016 is highly similar to that of the strain GII.P16/GII.2 [38]. Because the GII.P16/GII.2 caused a sharp increase in AGE outbreaks in many countries in Asia and Europe in the winter of 2016–2017, the major factors leading to the re-emergence of GII.P16/GII.2 and the increase of AGE outbreaks must be in the genome regions other than the capsid protein-encoding genes [10]. For example, the GII.P16 polymerase may confer the new strain/variant a better adaptability compared with the GII.P2 polymerase. As CaliciNet reported during 2016–2018, 81.2% of AGE outbreaks were caused by GII.P16/GII.2 in China [39].

Unlike GII.4 NoVs that caused AGE in people of all age groups, some previous reports have suggested that GII.3 is the predominant genotype in infants and children, implying a sustained, lifelong protection [40,41]. As found in our study, although the mean evolutionary rate of GII.3 (3.40 × 10^−3^–3.59 × 10^−3^ nucleotide substitutions/site/year) is comparable to other non-GII.4 genotypes, the amino acid variation of GII.3 VP1 was so little that no novel variant had emerged since 1970s. This may be the main reason for the low prevalence of GII.3 in recent years.

The first report of GII.6 NoVs was in 1971 in Henryton Hospital of Maryland, USA [42]. Our study identified three GII.6 variants (GII.6a, GII.6b and GII.6c) circulated since the 1970s, which is concordant with the results of earlier studies [43]. Although genotype GII.6 failed to present a linear relationship over time (*R*^2^ = 0.170), it showed a strong linear evolution trend (*R*^2^ = 0.88–0.98) when the three variants were analyzed separately, indicating a unique evolutionary mechanism [15]. So far, there have been only a few reports of AGE caused by GII.6 NoVs. For instance, GII.6 strains emerged as the second most prevailing strain after GII.4, causing AGE in children in Shizuoka, Japan, during 2008–2009 [44]. In addition, an AGE outbreak occurred in an elementary school in Shanghai in December of 2013 [45]. As reported in the United States from 2014 to 2015, 10.3% (94/910) of the NoV outbreaks were attributed to GII.6 NoVs [46]. Moreover, according to NoroNet, the NoV surveillance network, GII.6 accounted for 2.0–10.3% of NoV outbreaks and sporadic cases between 2005 and 2016 in Europe, Asia, Oceania and Africa [47]. Despite a lack of statistical significance in the year-to-year difference of GII.6-associated diseases, the large-scale epidemiological surveys and systematic reviews of NoV literatures showed that GII.6 was the second common genotype after GII.4.

The earliest GII.17 sequence available in the National Center for Biotechnology Information (NCBI) database was from a stool sample collected back in 1978 [48]. Since then, there were only a few reports of sporadic cases caused by GII.17 around the world and occasional detection of this genotype in environmental samples [49]. During the winter of 2014–2015, the novel GII.17 variant (designated variant D in this study) emerged to cause AGE outbreaks in East Asia [6]. As shown in a phylogenetic tree, pre-epidemic variant C and epidemic variant D of GII.17 shared a common ancestry and then diverged into two genetically distinct variants. Sang et al. [50] showed that their P2 subdomains are highly variable with 44% (56/128) amino acids variations, including two insertions at positions 295–296 and one deletion at position 385 (variants C and D) and one insertion at position 375 (variant D). These amino acid insertions and deletions may lead to the evasion of herd immunity and the change of population susceptibility, which may explain the high prevalence of GII.17 variant D.

There are several limitations in this study. Firstly, the virus RNA showed some degradation due to the long-term storage of stool specimens collected for NoV surveillance from Zhengding County, which made it difficult to detect NoVs. Secondly, most of the sequences were from GenBank database, which may not represent the actual status of NoV infections. However, we believed that the source of NoV sequences may not be as important as the molecular epidemiological trend and evolutionary patterns of NoV variants identified by our analysis. Furthermore, sequences from population-based surveillance in Zhengding County confirmed the reliability and consistency of results based on the sequences from GenBank database. Thirdly, due to the limitation of computer capabilities, only representative GII.4 sequences were selected for our analysis.

## 4. Materials and Methods

### 4.1. Dataset from Population-Based Diarrhea Surveillance

#### 4.1.1. Sample Collection

The time serial stool samples were collected from five surveillance groups of children under five years old in Zhengding County, Hebei Province, China, which covers the epidemic seasons from 2001 to 2019. The samples were 313, 338, 1091, 295 and 5363 for 2001–2002, 2004–2005, 2011–2012, 2016–2017 and 2018–2019, respectively. All bulk stools were stored at −80 °C until batch testing at the Key Laboratory of Medical Molecular Virology, Fudan University at the end of surveillance period.

#### 4.1.2. Complete VP1 Sequencing

Nucleic acid was extracted from stool supernatant by an automated bead-beating procedure using TianLong Stool DNA/RNA Extraction Kit (TianLong Science & Technology, Xi’an, China). The capsid protein-encoding regions of NoV genomes were amplified by conventional reverse transcription polymerase chain reaction (RT-PCR) using primers G1SKF/G1SKR and COG2F/G2SKR, as described previously [51]. All NoV-positive samples were selected for further sequencing of the complete VP1-encoding genes. RT was performed using GoScript™ Reverse Transcription System (Promega, Madison, WI, USA) with primer TX30SXN according to the manufacturer’s instructions. PCR to amplify a ~2.5kb cDNA fragment covering the complete VP1 and VP2-encoding genes of NoV genome were performed using PrimeSTAR^®^ HS DNA Polymerase (TaKaRa, Kusatsu, Japan) using a semi-nested PCR GII-specific primer set (GoG2F/Tx30SXN in the first-round PCR and G2SKF/Tx30SXN for the second-round PCR) for GII genogroup [52] and a one-step PCR GI-specific primer set (G1SKF/TX30SXN) for GI genogroup [53]. The cycling conditions included a denaturation step at 98 °C for 3 min; followed by 40 cycles of 98 °C for 10 s, 60 °C for 15 s and 72 °C for 3 min; followed by a final extension step at 72 °C for 10 min. All PCR products were purified and sequenced using Sanger dideoxy termination sequencing by the Biosune Co., Ltd. in Shanghai. After sequencing, the Basic Local Alignment Search Tool (BLAST) was applied to identify genotypes.

### 4.2. Dataset from GenBank

All available NoV sequences were retrieved by searching the corresponding taxonomy ID (142786) from human host in GenBank database (accessed on 1 January 2020). The sequences with sampling time and capsid protein genotyping information were screened and retained to analyze temporal distribution of NoV genotypes together with the sequences from our surveillance in Zhengding County.

### 4.3. Multiple Alignments and Phylogenetic Analyses

The complete VP1 sequences of representative genotypes (GII.2, GII.3, GII.4, GII.6 and GII.17) were extracted using ORFfinder [54] for phylogenetic analysis. Sequences of each genotype from population-based diarrhea surveillance and GenBank were separately aligned using tool MUSCLE [55] in the Molecular Evolutionary Genetics Analysis (MEGA) software (version 7) with default parameters [56]. A neighbor-joining (NJ) phylogenetic tree based on the nucleotide alignment of full-length ORF2 was reconstructed using a maximum composite likelihood model supported by bootstrap with 1000 replicates and visualized in MEGA v7. The divergence pattern and clock-likeness of the VP1-encoding nucleotide sequences were visualized by root-to-tip divergence plots constructed by TempEst v1.5.3 (formerly called Path-O-Gen) using phylogenetic tree and collection year of each strain [57]. The best-fitting root option was used to minimize the sum of the squared residuals to obtain the best correlation of the root-to-tip divergence. The nomenclature for the GII.4 variants was determined using an online NoV typing tool [58]. For non-GII.4 genotypes, variants were defined by a cutoff of 5% difference in amino acid sequences, combined with the phylogenetic tree [14].

### 4.4. Evolutionary Dynamic Analyses

In addition, the parameter values for the best-fit model of nucleotide substitution rates were determined to be GTR+G+I using the Akalike Information Criterion (AIC) as implemented in jModelTest v2.1.3 [59]. The Bayesian Markov Chain Monte Carlo (MCMC) approach as implemented in BEAST software v1.10.4 package [60] was used to jointly estimate the annual nucleotide replacement rate of each site (subs./site/year) and the time to the most recent common ancestor (TMRCA) of each genotype. Since no specified demographic model that showed the known cyclic annual/seasonal behavior of the organism was available, the Bayesian skyline model for population growth was selected as a flexible coalescent tree using a strict or relaxed molecular clock model in BEAST [61]. For all models, the MCMC was run until the convergence of all the parameters was confirmed by visual inspection and effective sample size (>200 in all parameters) using Tracer v1.7.1. The first 10% of logs from the MCMC were removed as a burn-in before the posterior values were summarized. Statistical uncertainty in the parameter estimates was given by the 95% highest probability density (HPD) intervals. Different clock models, a strict molecular clock, an uncorrelated log-normal model (UCLN), and an uncorrelated exponential derivation model (UCED) were compared in Tracer v.1.6 by calculating the Bayes factor (BF) with 1000 bootstraps under the posterior distribution. TreeAnnotator v1.8.4 and FigTree v1.4.3 were used to summarize the posterior tree distribution and to visualize the annotated maximum clade credibility tree, respectively.

The genetic distance between vaccine strain GII.4c and the current prevalent genotypes/variants was compared at an amino acid level using MEGA v7. The bivalent GI.1/GII.4c VLPs vaccine was developed by Takeda Pharmaceutical Company Limited. The vaccine strain GII.4c (GenBank accession: MK614455.1) [62], a consensus sequence deriving from GII.4 Houston_2002, Den_Haag_2006b and Yerseke_2006a strains was downloaded from GenBank.

### 4.5. Homologous Modeling

Three-dimensional structure of P domain was predicted by homology modeling using the Swiss Model server [63]. The model was built based on the published crystal structures of GII.4 NoV strain VA387 (PDB accession code: 2ORB) [64]. Residue changes in blocking epitopes between the vaccine strain GII.4c and the variant Sydney_2012 were mapped to the model by PyMol v1.3. Residue positions were numbered according to the reference strain (GenBank accession: JX459908).

## 5. Conclusions

A comprehensive understanding of the evolutionary patterns employed by different NoV genotypes is essential for vaccine design and development, including selection of suitable NoV strains as immunogen of vaccine candidates. In this study, Sydney_2012 variant of GII.4, variant A of GII.2, a strain of GII.3, variant B and C of GII.6 and variant D of GII.17 were preliminarily considered to be included in the immunogen of a candidate vaccine for broad efficacy. In addition, continual surveillance of future NoVs will be necessary to update the vaccine formula as needed.

## Figures and Tables

**Table 1:** Summary of the norovirus sequences that were analyzed in this study.

Genotypes	Variants	Sequences	Years	Time Span (Years)
GII.4	Bristol_1993	1	1987	1
	Camberwell_1994	11	1987–1988	2
	US95_96	13	1995–2004	10
	Farmington_Hills_2002	21	2002–2012	11
	Lanzhou_2002	3	2000–2002	3
	Asia_2003	27	2004–2011	8
	Kaiso_2003	2	2002–2003	2
	Hunter_2004	37	2002–2006	5
	Yerseke_2006a	34	2006–2015	10
	Den_Haag_2006b	56	2006–2015	10
	Apeldoorn_2007	50	2007–2009	3
	Cairo_2007	2	2007	1
	Osaka_2007	23	2005–2011	6
	New_Orleans_2009	45	2008–2013	6
	Sydney_2012	40	2010–2019	10
	Hong_Kong_2019	1	2019	1
GII.2	A	299	1971–2019	49
	B	2	2002	1
GII.3	-	139	1972–2019	48
GII.6	A	19	1971–2012	49
	B	18	1976–2019	44
	C	37	1977–2019	43
GII.17	A	8	1978–2016	39
	B	1	2005	1
	C	26	2013–2015	3
	D	460	2013–2019	7

**Table 2:** Nucleotide substitution rates and divergence times of norovirus VP1 genes over the past 30–50 years.

Genotypes	Years	No. of Sequences	Molecular Clock	Nucleotide Substitution Rate 10^−3^ Substitution/Site/Year (95% HPD)	TMRCA	
					No. of Years	Dates (Ranges)
GII.4	1987–2019	366	Strict Clock	4.95 (4.53–5.39)	38.5 (37.0–40.1)	1980.5 (1978.9–1982.0)
			UCLN	5.37 (4.84–5.88)	38.4 (34.7–43.5)	1980.6 (1975.5–1984.3)
			UCED	5.91 (5.33–6.52)	37.6 (33.7–43.2)	1981.4 (1975.8–1985.3)
GII.4 (Zhengding)	2002–2019	119	Strict Clock	1.55 (1.17–2.01)	51.2 (38.5–63.7)	1967.8 (1955.3–1980.5)
			UCLN	1.67 (1.10–2.52)	50.3 (29.5–70.4)	1968.7 (1948.6–1989.5)
			UCED	2.97 (1.48–4.43)	32.3 (17.9–56.1)	1986.7 (1962.9–2001.1)
GII.2	1971–2019	301	Strict Clock	2.30 (2.01–2.60)	88.3 (79.1–97.4)	1930.7 (1921.6–1939.9)
			UCLN	2.68 (2.19–3.20)	81.5 (60.5–105.6)	1937.5 (1913.4–1958.5)
			UCED	3.16 (2.52–3.76)	73.5 (55.0–99.3)	1945.5 (1919.7–1964.0)
GII.3	1972–2019	139	Strict Clock	3.40 (3.00–3.80)	51.0 (49.3–52.9)	1968.0 (1966.1–1969.7)
			UCLN	3.51 (2.99–4.04)	50.9 (47.3–55.1)	1968.1 (1963.9–1971.7)
			UCED	3.59 (2.99–4.21)	51.9 (47.0–60.1)	1967.1 (1958.9–1972.0)
GII.6	1971–2019	74	Strict Clock	2.39 (1.98–2.83)	163.4 (136.0–192.1)	1855.6 (1826.9–1883.0)
			UCLN	2.64 (1.96–3.34)	142.3 (96.9–189.6)	1876.7 (1829.4–1922.1)
			UCED	3.07 (1.86–4.28)	122.0 (68.1–193.7)	1897.0 (1825.3–1950.9)
GII.17	1978–2019	495	Strict Clock	1.54 (1.29–1.79)	227.1 (183.1–274.2)	1791.9 (1744.8–1835.9)
			UCLN	1.94 (1.26–2.65)	157.5 (67.5–276.0)	1861.5 (1743.0–1951.5)
			UCED	1.98 (1.37–2.60)	150.4 (74.5–253.4)	1868.6 (1765.6–1944.5)

Note: UCLN, uncorrelated lognormal relaxed clock. UCED, uncorrelated exponential relaxed clock. TMRCA, time to the most recent common ancestor. 95% HPD: 95% highest probability density.

**Table 3:** The amino acid distances among the main epidemic norovirus strains and vaccine strain GII.4c.

Variants	Vaccine GII.4c	GII.4 Sydney_2012	GII.2 Variant A	GII.3 Genotype	GII.6 Variant B	GII.6 Variant C
GII.4 Sydney_2012	0.053					
GII.2 Variant A	0.354	0.355				
GII.3 Genotype	0.319	0.338	0.299			
GII.6 Variant B	0.348	0.357	0.284	0.265		
GII.6 Variant C	0.342	0.352	0.301	0.261	0.076	
GII.17 Variant D	0.335	0.335	0.278	0.278	0.280	0.272

## Data Availability

The data that support the findings of this study are available from the corresponding author upon reasonable request.

## Supplementary Materials

The following are available online at https://www.mdpi.com/article/10.3390/pathogens10081012/s1, Table S1: The sequences of NoV GII.4 selected for analyses in this study.
