# Supplementary material for: Prevalence and Evolution of Noroviruses between 1966 and 2019, Implications for Vaccine Design
Source: Pathogens. 2021 Aug 11;10(8):1012. doi: 10.3390/pathogens10081012 (PMC8400007; doi:10.3390/pathogens10081012)
Supplement: Supplementary file 1 [file pathogens-10-01012-s001.zip › pathogens-1311973-supplementary.pdf]

**Supplementary Table S1. The sequences of NoV GII.4 selected for analyses in this study**

| Accession No. | Year | Country | Capsid subtype |
|---|---|---|---|
| FJ537137.1 | 1987 | USA | Bristol_1993 |
| JX289821.1 | 1987 | USA | Camberwell_1994 |
| KY424332.1 | 1987 | USA | Camberwell_1994 |
| KY424333.1 | 1987 | USA | Camberwell_1994 |
| KY424334.1 | 1987 | USA | Camberwell_1994 |
| KY424335.1 | 1987 | USA | Camberwell_1994 |
| KY424336.1 | 1987 | USA | Camberwell_1994 |
| KY424337.1 | 1987 | USA | Camberwell_1994 |
| KY424338.1 | 1987 | USA | Camberwell_1994 |
| FJ537136.1 | 1988 | USA | Camberwell_1994 |
| KY424339.1 | 1988 | USA | Camberwell_1994 |
| KY424340.1 | 1988 | USA | Camberwell_1994 |
| AB303922.1 | 1995 | NLD | US95_96 |
| FJ411169.1 | 1995 | USA | US95_96 |
| JQ478407.1 | 1997 | USA | US95_96 |
| DQ975270.1 | 1998 | JPN | US95_96 |
| AB303923.1 | 2000 | NLD | US95_96 |
| AB303924.1 | 2000 | NLD | US95_96 |
| AB303925.1 | 2000 | NLD | US95_96 |
| FJ411170.1 | 2000 | USA | Lanzou_2002 |
| AB504306.1 | 2001 | JPN | US95_96 |
| AB303926.1 | 2001 | NLD | US95_96 |
| AB303927.1 | 2001 | NLD | US95_96 |
| KJ407076.1 | 2001 | USA | US95_96 |
| Zhengding_38[#] | 2002 | CHN | Lanzou_2002 |
| FJ538900.1 | 2002 | FRA | Farmington_Hills_2002 |
| EU876890.1 | 2002 | FRA | Hunter_2004 |
| AB294778.1 | 2002 | JPN | US95_96 |
| AB303928.1 | 2002 | NLD | Farmington_Hills_2002 |
| AB303929.1 | 2002 | NLD | Kaiso_2003 |
| KU182476.1 | 2002 | TUN | Hunter_2004 |
| KU182477.1 | 2002 | TUN | Hunter_2004 |
| KU182478.1 | 2002 | TUN | Hunter_2004 |
| KU182479.1 | 2002 | TUN | Hunter_2004 |
| KU182480.1 | 2002 | TUN | Hunter_2004 |
| KU182481.1 | 2002 | TUN | Hunter_2004 |
| KU182482.1 | 2002 | TUN | Hunter_2004 |
| KU182483.1 | 2002 | TUN | Hunter_2004 |
| EU310927.1 | 2002 | USA | Lanzou_2002 |

| | | | |
|---|---|---|---|
| AB294780.1 | 2003 | JPN | Farmington_Hills_2002 |
| AB294779.1 | 2003 | JPN | Kaiso_2003 |
| AB303930.1 | 2003 | NLD | Farmington_Hills_2002 |
| AB303931.1 | 2003 | NLD | Farmington_Hills_2002 |
| AB303932.1 | 2003 | NLD | Farmington_Hills_2002 |
| AB303933.1 | 2003 | NLD | Farmington_Hills_2002 |
| JQ320073.1 | 2003 | USA | Farmington_Hills_2002 |
| JQ965810.1 | 2003 | USA | Farmington_Hills_2002 |
| JX445152.1 | 2004 | CAN | Farmington_Hills_2002 |
| HM802546.1 | 2004 | CHN | Asia_2003 |
| Zhengding_47[#] | 2004 | CHN | Asia_2003 |
| Zhengding_78[#] | 2004 | CHN | Farmington_Hills_2002 |
| HM802541.1 | 2004 | CHN | Hunter_2004 |
| EU780734.1 | 2004 | ESP | Hunter_2004 |
| AB294782.1 | 2004 | JPN | Asia_2003 |
| AB504307.1 | 2004 | JPN | Asia_2003 |
| AB504308.1 | 2004 | JPN | Asia_2003 |
| AB504309.1 | 2004 | JPN | Asia_2003 |
| LC016599.1 | 2004 | JPN | Asia_2003 |
| LC016600.1 | 2004 | JPN | Asia_2003 |
| LC016601.1 | 2004 | JPN | Asia_2003 |
| LC016602.1 | 2004 | JPN | Asia_2003 |
| LC016603.1 | 2004 | JPN | Asia_2003 |
| LC016604.1 | 2004 | JPN | Asia_2003 |
| LC016605.1 | 2004 | JPN | Asia_2003 |
| LC016606.1 | 2004 | JPN | Asia_2003 |
| LC016607.1 | 2004 | JPN | Asia_2003 |
| AB294781.1 | 2004 | JPN | Farmington_Hills_2002 |
| AB294783.1 | 2004 | JPN | Hunter_2004 |
| AB303934.1 | 2004 | NLD | Farmington_Hills_2002 |
| AB303935.1 | 2004 | NLD | Farmington_Hills_2002 |
| AB303936.1 | 2004 | NLD | Farmington_Hills_2002 |
| AB303937.1 | 2004 | NLD | Hunter_2004 |
| AB303938.1 | 2004 | NLD | Hunter_2004 |
| AB303939.1 | 2004 | NLD | Hunter_2004 |
| AB303940.1 | 2004 | NLD | Hunter_2004 |
| AB303941.1 | 2004 | NLD | Hunter_2004 |
| AY883096.1 | 2004 | NLD | Hunter_2004 |
| JX459596.1 | 2004 | SGP | Farmington_Hills_2002 |
| JX459595.1 | 2004 | SGP | Hunter_2004 |
| DQ658413.1 | 2004 | USA | Farmington_Hills_2002 |
| JQ478408.1 | 2004 | USA | Farmington_Hills_2002 |
| JQ798158.1 | 2004 | USA | Farmington_Hills_2002 |
| KC013592.1 | 2004 | USA | US95_96 |

| | | | |
|---|---|---|---|
| GU350225.1 | 2005 | CHN | Asia_2003 |
| HM802540.1 | 2005 | CHN | Hunter_2004 |
| AB294786.1 | 2005 | JPN | Asia_2003 |
| AB294787.1 | 2005 | JPN | Asia_2003 |
| AB504310.1 | 2005 | JPN | Asia_2003 |
| AB504313.1 | 2005 | JPN | Asia_2003 |
| AB504314.1 | 2005 | JPN | Asia_2003 |
| AB294784.1 | 2005 | JPN | Hunter_2004 |
| AB294785.1 | 2005 | JPN | Hunter_2004 |
| AB504311.1 | 2005 | JPN | Hunter_2004 |
| AB504312.1 | 2005 | JPN | Hunter_2004 |
| AB385628.1 | 2005 | NLD | Hunter_2004 |
| AB385629.1 | 2005 | NLD | Hunter_2004 |
| AB385635.1 | 2005 | NLD | Hunter_2004 |
| AB385636.1 | 2005 | NLD | Hunter_2004 |
| JX459597.1 | 2005 | SGP | Hunter_2004 |
| JX459598.1 | 2005 | SGP | Hunter_2004 |
| JF827296.1 | 2005 | USA | Hunter_2004 |
| FJ411171.1 | 2005 | USA | Osaka_2007 |
| EF684915.2 | 2006 | AUS | Den_Haag_2006b |
| JX445158.1 | 2006 | CAN | Den_Haag_2006b |
| JX445153.1 | 2006 | CAN | Hunter_2004 |
| JX445154.1 | 2006 | CAN | Hunter_2004 |
| JX445156.1 | 2006 | CAN | Yerseke_2006a |
| JX445157.1 | 2006 | CAN | Yerseke_2006a |
| HM802525.1 | 2006 | CHN | Den_Haag_2006b |
| HQ456322.1 | 2006 | CHN | Yerseke_2006a |
| EU876892.1 | 2006 | EGY | Den_Haag_2006b |
| EU876882.1 | 2006 | EGY | Osaka_2007 |
| EU876884.1 | 2006 | EGY | Osaka_2007 |
| EU876883.1 | 2006 | EGY | Yerseke_2006a |
| EU876885.1 | 2006 | EGY | Yerseke_2006a |
| EU876886.1 | 2006 | EGY | Yerseke_2006a |
| EU876895.1 | 2006 | FRA | Den_Haag_2006b |
| EU876894.1 | 2006 | FRA | Yerseke_2006a |
| AB294791.1 | 2006 | JPN | Asia_2003 |
| AB294792.1 | 2006 | JPN | Asia_2003 |
| AB447448.1 | 2006 | JPN | Asia_2003 |
| AB504315.1 | 2006 | JPN | Asia_2003 |
| AB504316.1 | 2006 | JPN | Asia_2003 |
| AB504321.1 | 2006 | JPN | Den_Haag_2006b |
| AB294788.1 | 2006 | JPN | Hunter_2004 |
| AB294789.1 | 2006 | JPN | Yerseke_2006a |
| AB294790.1 | 2006 | JPN | Yerseke_2006a |

| | | | |
|---|---|---|---|
| AB447432.1 | 2006 | JPN | Yerseke_2006a |
| AB447433.1 | 2006 | JPN | Yerseke_2006a |
| AB447458.1 | 2006 | JPN | Yerseke_2006a |
| EF126966.1 | 2006 | NLD | Den_Haag_2006b |
| EF126961.1 | 2006 | NLD | Hunter_2004 |
| EF126962.1 | 2006 | NLD | Hunter_2004 |
| AB385632.1 | 2006 | NLD | Yerseke_2006a |
| AB385633.1 | 2006 | NLD | Yerseke_2006a |
| AB385637.1 | 2006 | NLD | Yerseke_2006a |
| AB385638.1 | 2006 | NLD | Yerseke_2006a |
| AB385639.1 | 2006 | NLD | Yerseke_2006a |
| AB385643.1 | 2006 | NLD | Yerseke_2006a |
| EF126963.1 | 2006 | NLD | Yerseke_2006a |
| EF126964.1 | 2006 | NLD | Yerseke_2006a |
| EF187497.2 | 2006 | NZL | Yerseke_2006a |
| JX459613.1 | 2006 | SGP | Den_Haag_2006b |
| JX459599.1 | 2006 | SGP | Hunter_2004 |
| JX459607.1 | 2006 | SGP | Yerseke_2006a |
| JX459612.1 | 2006 | SGP | Yerseke_2006a |
| HQ693090.1 | 2006 | THA | Den_Haag_2006b |
| JQ478409.1 | 2006 | USA | Den_Haag_2006b |
| FJ411172.1 | 2006 | USA | Osaka_2007 |
| GQ845368.2 | 2007 | AUS | Cairo_2007 |
| JQ613516.1 | 2007 | AUS | Den_Haag_2006b |
| JQ613511.1 | 2007 | AUS | Yerseke_2006a |
| JQ613517.1 | 2007 | AUS | Yerseke_2006a |
| GQ856445.1 | 2007 | CHN | Den_Haag_2006b |
| EU876888.1 | 2007 | EGY | Cairo_2007 |
| EU876887.1 | 2007 | EGY | Yerseke_2006a |
| EU876889.1 | 2007 | EGY | Yerseke_2006a |
| KR131762.1 | 2007 | IND | Den_Haag_2006b |
| KR131764.1 | 2007 | IND | Osaka_2007 |
| KR131770.1 | 2007 | IND | Osaka_2007 |
| AB665364.1 | 2007 | JPN | Den_Haag_2006b |
| AB541190.1 | 2007 | JPN | Osaka_2007 |
| AB541319.1 | 2007 | JPN | Osaka_2007 |
| AB541321.1 | 2007 | JPN | Osaka_2007 |
| AB541323.1 | 2007 | JPN | Osaka_2007 |
| AB541325.1 | 2007 | JPN | Osaka_2007 |
| AB541267.1 | 2007 | JPN | Yerseke_2006a |
| JQ622197.1 | 2007 | KOR | Den_Haag_2006b |
| AB445395.1 | 2007 | NLD | Apeldoorn_2007 |
| AB385640.1 | 2007 | NLD | Yerseke_2006a |
| JX459628.1 | 2007 | SGP | Den_Haag_2006b |

| | | | |
|---|---|---|---|
| JX459621.1 | 2007 | SGP | Yerseke_2006a |
| AB496912.1 | 2007 | ZAF | Yerseke_2006a |
| JQ613524.1 | 2008 | AUS | Den_Haag_2006b |
| GQ845367.2 | 2008 | AUS | New_Orleans_2009 |
| GQ845369.3 | 2008 | AUS | Osaka_2007 |
| GQ413969.1 | 2008 | AUS | Osaka_2007 |
| GQ849126.1 | 2008 | AUS | Yerseke_2006a |
| JX445161.1 | 2008 | CAN | Apeldoorn_2007 |
| JX445162.1 | 2008 | CAN | Den_Haag_2006b |
| HQ456341.1 | 2008 | CHN | Apeldoorn_2007 |
| GQ856457.1 | 2008 | CHN | Den_Haag_2006b |
| KJ144999.1 | 2008 | DNK | Den_Haag_2006b |
| GQ246791.1 | 2008 | FRA | Apeldoorn_2007 |
| GQ246793.1 | 2008 | FRA | Apeldoorn_2007 |
| GQ246794.1 | 2008 | FRA | Apeldoorn_2007 |
| GQ246797.1 | 2008 | FRA | Apeldoorn_2007 |
| EU876891.1 | 2008 | FRA | Den_Haag_2006b |
| KR131772.1 | 2008 | IND | Apeldoorn_2007 |
| KR131773.1 | 2008 | IND | New_Orleans_2009 |
| KR131763.1 | 2008 | IND | Osaka_2007 |
| KR131765.1 | 2008 | IND | Osaka_2007 |
| KR131766.1 | 2008 | IND | Osaka_2007 |
| KR131767.1 | 2008 | IND | Osaka_2007 |
| KR131771.1 | 2008 | IND | Osaka_2007 |
| AB541198.1 | 2008 | JPN | Apeldoorn_2007 |
| AB541202.1 | 2008 | JPN | Apeldoorn_2007 |
| AB541268.1 | 2008 | JPN | Apeldoorn_2007 |
| AB541272.1 | 2008 | JPN | Apeldoorn_2007 |
| AB541274.1 | 2008 | JPN | Apeldoorn_2007 |
| AB541310.1 | 2008 | JPN | Apeldoorn_2007 |
| AB541312.1 | 2008 | JPN | Apeldoorn_2007 |
| AB541318.1 | 2008 | JPN | Apeldoorn_2007 |
| AB541320.1 | 2008 | JPN | Apeldoorn_2007 |
| AB541322.1 | 2008 | JPN | Apeldoorn_2007 |
| AB541360.1 | 2008 | JPN | Den_Haag_2006b |
| GU390901.1 | 2008 | KOR | Apeldoorn_2007 |
| GU390902.1 | 2008 | KOR | Apeldoorn_2007 |
| HQ009513.1 | 2008 | KOR | Apeldoorn_2007 |
| GU390900.1 | 2008 | KOR | Den_Haag_2006b |
| JX459630.1 | 2008 | SGP | Apeldoorn_2007 |
| JX459634.1 | 2008 | SGP | Den_Haag_2006b |
| AB492092.1 | 2008 | SWE | Apeldoorn_2007 |
| JN183166.1 | 2008 | SWE | Den_Haag_2006b |
| GU270580.1 | 2008 | USA | Apeldoorn_2007 |

| | | | |
|---|---|---|---|
| KC990829.1 | 2008 | USA | Den_Haag_2006b |
| GQ850882.1 | 2008 | USA | Osaka_2007 |
| HM748973.2 | 2009 | AUS | Apeldoorn_2007 |
| JQ613529.1 | 2009 | AUS | Apeldoorn_2007 |
| JQ613525.1 | 2009 | AUS | Den_Haag_2006b |
| JQ613570.1 | 2009 | AUS | New_Orleans_2009 |
| JX445163.1 | 2009 | CAN | Den_Haag_2006b |
| HQ005292.1 | 2009 | CHN | Apeldoorn_2007 |
| HM590583.1 | 2009 | CHN | Den_Haag_2006b |
| HQ005293.1 | 2009 | CHN | New_Orleans_2009 |
| GQ246795.1 | 2009 | FRA | Apeldoorn_2007 |
| GQ246796.1 | 2009 | FRA | Apeldoorn_2007 |
| GQ246798.1 | 2009 | FRA | Apeldoorn_2007 |
| GQ246799.1 | 2009 | FRA | Den_Haag_2006b |
| GQ246801.1 | 2009 | FRA | New_Orleans_2009 |
| GQ246800.1 | 2009 | FRA | Osaka_2007 |
| KR131774.1 | 2009 | IND | New_Orleans_2009 |
| KR131768.1 | 2009 | IND | Osaka_2007 |
| KR131769.1 | 2009 | IND | Osaka_2007 |
| AB933729.1 | 2009 | JPN | Apeldoorn_2007 |
| AB933730.1 | 2009 | JPN | Apeldoorn_2007 |
| AB933656.1 | 2009 | JPN | Den_Haag_2006b |
| AB933757.1 | 2009 | JPN | New_Orleans_2009 |
| HM635099.2 | 2009 | KOR | Apeldoorn_2007 |
| HM635100.2 | 2009 | KOR | Apeldoorn_2007 |
| HM635101.2 | 2009 | KOR | Apeldoorn_2007 |
| HM635155.2 | 2009 | KOR | Apeldoorn_2007 |
| HM635156.2 | 2009 | KOR | Apeldoorn_2007 |
| JX459638.1 | 2009 | SGP | Apeldoorn_2007 |
| JX459648.1 | 2009 | SGP | Den_Haag_2006b |
| JX459650.1 | 2009 | SGP | New_Orleans_2009 |
| KX354088.1 | 2009 | USA | Den_Haag_2006b |
| KX353954.1 | 2009 | USA | New_Orleans_2009 |
| KC960615.1 | 2009 | VNM | Den_Haag_2006b |
| JQ613572.1 | 2010 | AUS | Den_Haag_2006b |
| JQ613573.1 | 2010 | AUS | New_Orleans_2009 |
| JX445166.1 | 2010 | CAN | New_Orleans_2009 |
| JF713047.1 | 2010 | CHN | Den_Haag_2006b |
| HM191773.1 | 2010 | CHN | New_Orleans_2009 |
| HF952131.1 | 2010 | GBR | New_Orleans_2009 |
| HM625866.1 | 2010 | HUN | New_Orleans_2009 |
| KR131776.1 | 2010 | IND | New_Orleans_2009 |
| KP244313.1 | 2010 | ITA | New_Orleans_2009 |
| LC153749.1 | 2010 | JPN | Den_Haag_2006b |

| | | | |
|---|---|---|---|
| AB933745.1 | 2010 | JPN | New_Orleans_2009 |
| JX448566.1 | 2010 | KOR | Apeldoorn_2007 |
| HM635103.2 | 2010 | KOR | New_Orleans_2009 |
| KF060124.1 | 2010 | NZL | Sydney_2012 |
| JX459655.1 | 2010 | SGP | Den_Haag_2006b |
| JX459657.1 | 2010 | SGP | New_Orleans_2009 |
| KF429782.1 | 2010 | USA | Apeldoorn_2007 |
| KX354090.1 | 2010 | USA | Den_Haag_2006b |
| KX354052.1 | 2010 | USA | New_Orleans_2009 |
| KC960616.1 | 2010 | VNM | New_Orleans_2009 |
| JX459903.1 | 2011 | AUS | Apeldoorn_2007 |
| JX459906.1 | 2011 | AUS | Den_Haag_2006b |
| KF060121.1 | 2011 | AUS | New_Orleans_2009 |
| JX445168.1 | 2011 | CAN | New_Orleans_2009 |
| KF509947.2 | 2011 | CAN | Sydney_2012 |
| JX644033.1 | 2011 | CHN | Den_Haag_2006b |
| Zhengding_12[#] | 2011 | CHN | Den_Haag_2006b |
| JX644034.1 | 2011 | CHN | New_Orleans_2009 |
| Zhengding_41[#] | 2011 | CHN | New_Orleans_2009 |
| HF952135.1 | 2011 | GBR | New_Orleans_2009 |
| KR131782.1 | 2011 | IND | New_Orleans_2009 |
| KP244320.1 | 2011 | ITA | New_Orleans_2009 |
| KF668567.1 | 2011 | ITA | Sydney_2012 |
| AB933759.1 | 2011 | JPN | Apeldoorn_2007 |
| AB933728.1 | 2011 | JPN | Asia_2003 |
| AB972501.1 | 2011 | JPN | Den_Haag_2006b |
| AB972500.1 | 2011 | JPN | New_Orleans_2009 |
| AB972505.1 | 2011 | JPN | Sydney_2012 |
| KJ541743.1 | 2011 | KOR | Den_Haag_2006b |
| JX459659.1 | 2011 | SGP | New_Orleans_2009 |
| KF429791.1 | 2011 | USA | Apeldoorn_2007 |
| KX354073.1 | 2011 | USA | Den_Haag_2006b |
| KX608856.1 | 2011 | USA | New_Orleans_2009 |
| KX608858.1 | 2011 | USA | Osaka_2007 |
| KX354057.1 | 2011 | USA | Sydney_2012 |
| KC631814.1 | 2011 | USA | Yerseke_2006a |
| KC631815.1 | 2011 | USA | Yerseke_2006a |
| KP784693.1 | 2011 | ZAF | New_Orleans_2009 |
| KF060120.1 | 2012 | AUS | New_Orleans_2009 |
| KF177449.1 | 2012 | AUS | Sydney_2012 |
| KU311162.1 | 2012 | CAN | Den_Haag_2006b |
| JX445169.1 | 2012 | CAN | New_Orleans_2009 |
| KF509946.3 | 2012 | CAN | Sydney_2012 |
| KC517365.1 | 2012 | CHN | Den_Haag_2006b |

| | | | |
|---|---|---|---|
| Zhengding_1055# | 2012 | CHN | Den_Haag_2006b |
| KC517363.1 | 2012 | CHN | New_Orleans_2009 |
| JX629458.1 | 2012 | CHN | Sydney_2012 |
| KM406485.1 | 2012 | FRA | Sydney_2012 |
| KR131779.1 | 2012 | IND | New_Orleans_2009 |
| KP244322.1 | 2012 | ITA | New_Orleans_2009 |
| KF668568.1 | 2012 | ITA | Sydney_2012 |
| AB974408.1 | 2012 | JPN | Den_Haag_2006b |
| AB972515.1 | 2012 | JPN | New_Orleans_2009 |
| LC101819.1 | 2012 | JPN | Sydney_2012 |
| KM272334.1 | 2012 | KOR | Sydney_2012 |
| LN854565.1 | 2012 | NLD | Den_Haag_2006b |
| LN854572.1 | 2012 | NLD | New_Orleans_2009 |
| KF060153.1 | 2012 | NZL | Sydney_2012 |
| KF768522.1 | 2012 | SWE | New_Orleans_2009 |
| KF429768.1 | 2012 | USA | Apeldoorn_2007 |
| KF712493.1 | 2012 | USA | Apeldoorn_2007 |
| KF712494.1 | 2012 | USA | Apeldoorn_2007 |
| KF712499.1 | 2012 | USA | Apeldoorn_2007 |
| KF712500.1 | 2012 | USA | Apeldoorn_2007 |
| KF712506.1 | 2012 | USA | Apeldoorn_2007 |
| KF712507.1 | 2012 | USA | Apeldoorn_2007 |
| KX354084.1 | 2012 | USA | Den_Haag_2006b |
| JX126912.1 | 2012 | USA | Farmington_Hills_2002 |
| JX126913.1 | 2012 | USA | Farmington_Hills_2002 |
| KF429762.1 | 2012 | USA | New_Orleans_2009 |
| KY424330.1 | 2012 | USA | Sydney_2012 |
| KX354001.1 | 2012 | VIR | Den_Haag_2006b |
| LC177653.1 | 2012 | VNM | Den_Haag_2006b |
| LC177654.1 | 2012 | VNM | New_Orleans_2009 |
| KP784698.1 | 2012 | ZAF | New_Orleans_2009 |
| KP784697.1 | 2012 | ZAF | Sydney_2012 |
| KF177437.1 | 2013 | AUS | Sydney_2012 |
| KU311163.1 | 2013 | CAN | Den_Haag_2006b |
| KF306214.1 | 2013 | CHN | Sydney_2012 |
| KR131784.1 | 2013 | IND | Sydney_2012 |
| KP244317.1 | 2013 | ITA | New_Orleans_2009 |
| KF378731.1 | 2013 | JPN | Sydney_2012 |
| KX354091.1 | 2013 | USA | Den_Haag_2006b |
| KF712502.1 | 2013 | USA | New_Orleans_2009 |
| KX354112.1 | 2013 | USA | Sydney_2012 |
| LC177656.1 | 2013 | VNM | Sydney_2012 |
| KU311158.1 | 2014 | CAN | Sydney_2012 |
| KJ649701.1 | 2014 | CHN | Sydney_2012 |

| | | | |
|---|---|---|---|
| LC018709.1 | 2014 | JPN | Sydney_2012 |
| LN854566.1 | 2014 | NLD | Sydney_2012 |
| KX354134.1 | 2014 | USA | Sydney_2012 |
| LC177657.1 | 2014 | VNM | Sydney_2012 |
| KP864107.1 | 2015 | CHN | Sydney_2012 |
| KY341923.1 | 2015 | HUN | Sydney_2012 |
| LC066046.2 | 2015 | JPN | Sydney_2012 |
| MG786781.1 | 2015 | THA | Sydney_2012 |
| KX354128.1 | 2015 | USA | Den_Haag_2006b |
| KX907727.1 | 2015 | USA | Sydney_2012 |
| LC177661.1 | 2015 | VNM | Sydney_2012 |
| KX767083.1 | 2016 | AUS | Sydney_2012 |
| KU678203.1 | 2016 | CHN | Sydney_2012 |
| Zhengding_4[#] | 2016 | CHN | Sydney_2012 |
| LC325217.1 | 2016 | JPN | Sydney_2012 |
| MK073894.1 | 2016 | USA | Sydney_2012 |
| KY628449.1 | 2017 | CHN | Sydney_2012 |
| LC331997.1 | 2017 | JPN | Sydney_2012 |
| MN400355.2 | 2019 | CHN | Hong_Kong_2019 |

Note: # The sequences were from population-based diarrhea surveillance conducted in Zhengding county, Hebei province.
